# Incidence and Risk Factors for Venous Thromboembolism in Female Patients Undergoing Breast Surgery

**DOI:** 10.3390/cancers14040988

**Published:** 2022-02-16

**Authors:** Ambrogio P. Londero, Serena Bertozzi, Carla Cedolini, Silvia Neri, Michela Bulfoni, Maria Orsaria, Laura Mariuzzi, Alessandro Uzzau, Andrea Risaliti, Giovanni Barillari

**Affiliations:** 1 Academic Unit of Obstetrics and Gynaecology, Department of Neuroscience, Rehabilitation, Ophthalmology, Genetics, Maternal and Infant Health, University of Genoa, 16132 Genova, Italy; 2 Ennergi Research (Non-Profit Organisation), 33050 Lestizza, Italy; 3 Breast Unit, University Hospital of Udine, 33100 Udine, Italy; carla.cedolini@asufc.sanita.fvg.it; 4 Clinic of Surgery, University Hospital of Udine, 33100 Udine, Italy; neri.silvia@spes.uniud.it (S.N.); andrea.risaliti@uniud.it (A.R.); 5 Institute of Pathologic Anatomy, University Hospital of Udine, 33100 Udine, Italy; michela.bulfoni@uniud.it (M.B.); maria.orsaria@asufc.sanita.fvg.it (M.O.); laura.mariuzzi@uniud.it (L.M.); 6 Department of Medical Area (DAME), University of Udine, 33100 Udine, Italy; alessandro.uzzau@uniud.it; 7 Center for Hemorrhagic and Thrombotic Diseases, ASUFC “Santa Maria della Misericordia”, 33100 Udine, Italy; barillarigiovanni@yahoo.it

**Keywords:** breast cancer, venous thromboembolism, deep venous thrombosis, pulmonary embolism, basal-like subtype, comedo-like necrosis

## Abstract

**Simple Summary:**

Despite the known multiple correlations between venous thromboembolism (VTE) and breast cancer, only a limited number of studies have investigated the association between breast cancer and VTE during the long-term follow-up. Given the constantly increasing number of newly diagnosed breast cancers, the significance of focusing research on the correlation between breast cancer and VTE is becoming increasingly relevant. Furthermore, VTE has a high impact on a patient’s quality of life, with permanent consequences or mortality in the most severe cases. Thus, this study aims to assess the occurrence and timing of VTE during a long period of follow-up to analyze possible correlated factors and the overall survival. These results could impact the health care community, adding knowledge about VTE risk factors. These factors can be helpful as prognostic information and eventually target preventive treatment for VTE because the co-existence of invasive breast cancer VTE has a substantial impact on survival.

**Abstract:**

Albeit it does not have the highest venous thromboembolism (VTE) incidence compared to other neoplasms, breast cancer contributes to many VTE events because it is the most diagnosed tumor in women. We aim to analyze the occurrence and timing of VTE during the follow-up of patients who underwent breast surgery, the possible correlated factors, and the overall survival. This retrospective study included all female patients diagnosed with mammary pathology and surgically treated in our clinic between January 2002 and January 2012. Of 5039 women who underwent breast surgery, 1056 were found to have no evidence of malignancy, whereas 3983 were diagnosed with breast cancer. VTE rate resulted significantly higher in patients with invasive breast cancer than in women with benign breast disease or carcinoma in situ. Invasive cancers other than lobular or ductal were associated with a higher VTE rate. In addition, chronic hypertension, high BMI, cancer type, and evidence of metastasis turned out to be the most significant risk factors for VTE in women who underwent breast surgery. Moreover, VTE occurrence significantly impacted survival in invasive breast cancer patients. Compared to women with benign mammary pathology, VTE prevalence in women with breast cancer is significantly higher. The knowledge about the risk factors of VTE could be helpful as prognostic information, but also to eventually target preventive treatment strategies for VTE, as far as the co-existence of invasive breast cancer and VTE has a significantly negative impact on survival.

## 1. Introduction

Breast cancer is the most common female cancer worldwide, being 25% of new female cancer cases and the first cancer-related mortality cause (16% of all deaths) [1]. Globally, cancer among women is associated with a significant burden of venous thromboembolism (VTE). Despite the low incidence of VTE in localized breast cancer (5 per 1000 person years), the great number of early breast cancers worldwide justifies the numerous VTE cases associated with breast cancer (about 14% of all cancer-associated VTE) [2,3]. In particular, the introduction of systematic mammographic screening programs all over the world increased the number of early breast cancer diagnoses, as well as the number of women susceptible to surgical treatment, and significantly improved the survival after surgery or after the exposure to adjuvant treatments [4,5]. 

The correlation between cancer and VTE has been known for over a century (since 1864) [6], and the acquired thrombophilic state related to malignancies has been thoroughly analyzed in multiple studies [7,8,9,10]. In fact, there is a recognized VTE risk linked to the surgical treatment of the neoplasm [11], a hazard related to adjuvant therapies [5,12,13], and an intrinsic VTE risk linked to the possible association between blood hypercoagulability and tumor aggressiveness [7,14]. 

Despite these evident multiple correlations between VTE and breast cancer, only a limited number of studies have investigated the association between breast cancer and VTE or pulmonary embolism (PE) during the long-term follow-up [11,14]. However, given the constantly increasing number of newly diagnosed breast cancers, the importance of focusing research on the correlation between breast cancer and VTE is becoming increasingly relevant. Furthermore, VTE and PE are both pathologies with a high impact on a patient’s quality of life, with permanent consequences or mortality in the most severe cases [11,14]. Hence, accurate knowledge of the subject and the possible risk factors in women undergoing treatment for breast cancer is necessary, including the risk associated with breast surgery alone than the surgical treatment of the neoplastic pathology. 

This study aims to assess the occurrence and timing of VTE during a 10 year follow-up period in female patients who underwent breast surgery to analyze possible correlated factors and the overall survival.

## 2. Materials and Methods

This retrospective chart review study considered all women who underwent breast surgery in our center between January 2002 and January 2012. This study was conducted according to the Declaration of Helsinki. It followed the dictates of the general authorization to process personal data for scientific research purposes by the Italian Data Protection Authority. All consecutive women treated by breast surgery during this period were included in the present study. All male cases treated by breast surgery were excluded. Information was gathered from the clinical files of our center by medical doctor experts in breast surgery supervised by a specialist in thrombosis and hemostasis. 

In this study, we considered the main composite outcome the occurrence of objectively documented VTE episodes during the follow-up period. Secondary outcomes were the possible predictive factors for VTE occurrence and the overall survival in invasive breast cancer. A VTE event was defined as “surgery-related” any time its occurrence happened within the first three months after breast surgery [15]. In this study, we considered as VTE the presence of deep vein thrombosis (DVT) or pulmonary embolism (PE), confirmed by objective tests. DVT was objectively verified using standard imaging techniques, including compression ultrasonography, computer tomography venography, or magnetic resonance venography [16]. Meanwhile, PE was documented by contrast-enhanced computed tomography or ventilation-perfusion scintigraphy [16]. In addition, we considered the follow-up time from the first breast surgery to the last known outpatient visit.

Evaluating all women treated with breast surgery, we collected the following information: women’s age, body mass index (BMI), tobacco smoke, family history of breast or ovarian cancer, current or previous use of estrogen–progestin drugs, post-menopausal status, history of previous VTE, hypothyroidism, diabetes mellitus, chronic arterial hypertension, chronic lung disease, chronic heart failure, type of breast surgery, type of axilla surgery, and definitive histological results. In the case of invasive or in situ carcinoma, we collected the following additional information: tumor size, nodal status, TNM stage, and tumor grading, the presence of comedo-like necrosis, multifocality/multicentricity, extensive intraductal component (EIC), peritumoral vascular invasion (PVI), and peritumoral inflammation, breast cancer molecular subtype, lymph node characteristics (e.g., presence of isolated tumor cells, micrometastases, extracapsular lymph node invasion, or matted axilla lymph nodes), non-surgical treatments (e.g., neo-adjuvant or adjuvant chemotherapy), and the eventual occurrence of loco-regional or distant recurrences. 

Women with hypothyroidism included those undergoing thyroxine replacement therapy. Women affected by diabetes mellitus were those treated with oral anti-diabetic drugs or insulin. In this study, women suffering from chronic hypertension regularly used anti-hypertensive medications. Chronic lung diseases included chronic obstructive pulmonary diseases (e.g., chronic bronchitis, emphysema, asthma, bronchiectasis, or cystic fibrosis) and chronic restrictive lung diseases (e.g., idiopathic pulmonary fibrosis or other interstitial lung diseases, sarcoidosis, and neuromuscular diseases). Patients affected by symptomatic chronic heart failure were considered those with an objective test confirmation. 

As far as breast cancer was concerned, tumor stage was defined according to the VII edition of the TNM classification (AJCC/UICC) and tumor histology according to the World Health Organization criteria [17,18]. Furthermore, the tumor grade was evaluated following Elston and Ellis recommendations [17]. In this study, molecular subtypes of breast cancer were evaluated as previously described [19]. Moreover, the presence of PVI was considered according to Rosen and Oberman criteria, as previously stated [17]. Additionally, the expression and quantification of estrogen receptor, progesterone receptor, Her-2/Neu, and the proliferative tumor fraction (Mib1/Ki67) were evaluated as previously described [19]. In addition, the lymph node extracapsular invasion was defined as the extracapsular growth of tumor cells, invasion of perinodal fat or extranodal location of tumor cells [19]. Furthermore, the finding of hardened lymph nodes, increased in diameter and fixed to each other, was defined as matted lymph nodes. 

Surgical removal of the breast lesion was performed with breast-conserving surgery or mastectomy, followed by breast oncoplastic surgery or immediate breast reconstruction when appropriate. Non-palpable breast lesions were removed by wire hook localization or radio-guided occult lesion localization as previously described [20,21]. In all cases without any evident clinical involvement of axillary lymph nodes, the sentinel lymph node biopsy was performed as previously described [19,22]. 

After all surgical procedures for breast carcinoma, a standard VTE prophylaxis was prescribed, based on low molecular weight heparin. Low molecular weight heparin was subcutaneously administered, at a dosage adjusted for weight (0.5 mg/kg) and eventually additive VTE risk factors, for 28 consecutive days starting from the evening of the surgery date. Because of the non-interventional nature of the study, no predefined therapeutic protocol was adopted in the case of a VTE event, and treatment decisions were left at the treating physician’s discretion. Usually, in the case of VTE, low molecular weight heparin was administered subcutaneously twice daily for 6 weeks at a dosage based on patient weight; additional vitamin K antagonists or thrombolytic agents were administered when appropriate [16]. 

Data analysis was performed using R (version 3.6.3, R Core Team, Vienna, Austria) and considering a *p*-value < 0.05 as significant. Missing data were considered as NA. In addition, we excluded all variables with more than 40% missing values from the multivariate analysis. Univariate analysis was performed by Fisher exact test or chi-square test in the case of categorical variables, and Wilcoxon test or t-test in the case of continuous variables. We also conducted a Kaplan–Meier analysis and drew cumulative events curves. A competing risk model in the case of breast cancer was also used to assess the cumulative events [23]. Univariate and multivariate Cox proportional hazards model analyses were also performed, considering the occurrence of DVT, PE and VTE as the dependent variables in separate models. In the initial multivariate Cox proportional hazards model, all variables were introduced as covariates that were seen to have a *p*-value of less than 0.200 at the univariate investigation. Interaction terms were tested in the Cox regression models and excluded from the model if non-significant. Similarly, univariate and multivariate Cox proportional hazards model analyses were also performed for overall survival, considering VTE a time-dependent covariate. The Grambsch and Therneau test was used to assess the proportional hazard assumption for the Cox regression models [24].

## 3. Results

### 3.1. Population Description

This study included all consecutive 5039 patients who underwent breast surgery during the considered period. Among the included women, 20.96% (1056/5039) resulted in having benign histology, 9.11% (459/5039) ductal carcinoma in situ, 53.48% (2695/5039) invasive ductal carcinoma, 8.91% (449/5039) invasive lobular carcinoma, 4.54% (229/5039) ductal and lobular invasive carcinoma, and 3.00% (151/5039) different types of invasive carcinoma other than ductal or lobular. 

The mean age of our population at surgery was 57.72 years (±14.27), the mean BMI was 25.87 kg/m^2^ (±3.94), and the median follow-up was 75 months (IQR 47–114). Table 1 shows the population characteristics. Of the whole population, 1.61% of the women (81/5039) presented a positive history of previous VTE and 26.35% (1328/5039) chronic hypertension. Table 1B shows the characteristics of women affected by breast cancer. Adjuvant chemotherapy was administered to 39.76% of women (1401/3524), while hormonal therapy was given to 74.32% (2619/3524) of them. In particular, tamoxifen was chosen in 1282 of these last 2619 women (48.95%). 

Considering the TNM classification, the majority of cases had a T1 tumor size (62.7%), whereas a T2 tumor was present in 21.04% of the women (838/3983), T3 in 2.21% (88/3983), and T4 in 2.49% (99/3983) (Table S1). Furthermore, in the majority of cases, nodal status was N0 (69.65%, 2774/3983 women affected by breast cancer), follsuowed by N1 in 18.75% (747/3983), N2 in 6.18% (246/3983), and N3 in 5.42% (216/3983) (Table S1). Further characteristics specific to invasive breast cancers are reported in Table 1B. 

The TNM stage of women affected by breast cancer resulted in stage 0 in 11.55% (460/3983), stage I in 46.55% (1854/3983), stage II in 27.12% (1080/3983), stage III in 12.58% (501/3983), and stage IV in 2.21% (88/3983) of patients. Furthermore, another 247 women developed new distant metastases during the follow-up period with a prevalence of 7.01% (247/3524) (Table 1B).

### 3.2. VENOUS Thromboembolism Occurrence

**Figure 1:**
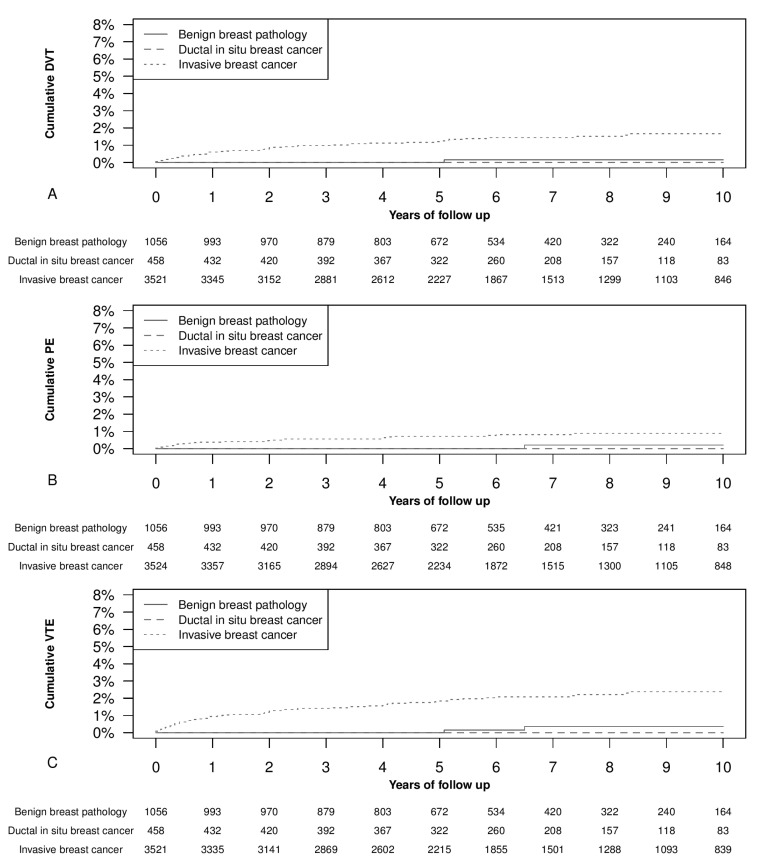
Occurrence of new venous thromboembolism (VTE) events in benign breast pathology, ductal carcinoma in situ breast, and invasive breast cancer. Panel (**A)** shows new deep vein thrombosis (DVT) events, with a significantly higher occurrence of DVT in invasive breast cancer than in carcinoma in situ (*p* < 0.05) and benign breast pathology (*p* < 0.05). Panel (**B)** shows new pulmonary embolism (PE) events, with a higher occurrence of PE in invasive breast cancer than in carcinoma in situ (*p* = 0.063) and benign breast pathology (*p* < 0.05). Panel (**C)** shows new VTE events with a significantly higher occurrence of VTE in invasive breast cancer than in carcinoma in situ (*p* < 0.05) and benign breast pathology (*p* < 0.05). The *p*-values refer to log-rank test.

Our main focus in this study was the occurrence of new VTE events. We observed thromboembolic events in 76 women during follow-up, and none of these occurred in women affected by ductal carcinoma in situ. In particular, we registered 54 new diagnoses of VTE and 28 new diagnoses of PE. In six cases, DVT and PE were coexistent. Figure 1 shows the cumulative events of VTE and PE. We found a significantly higher occurrence of new events in patients with invasive breast cancer than in those with carcinoma in situ or benign pathology. 

In detail, the surgery-related VTE events distribution within the first three months of follow-up was as follows. The cumulative DVT events at three months were 0% among patients with benign histology or ductal carcinoma in situ, and 0.23% (95% CI 0.07–0.38%) among those with invasive breast cancer (*p* < 0.05). The cumulative three months PE events were 0% among patients with benign histology or ductal carcinoma in situ, and 0.17% (95% CI 0.03–0.31%) among those with invasive breast cancer (*p* < 0.05). 

The cumulative three months VTE events were 0% among women with benign histology or ductal carcinoma in situ, and 0.4% (95% CI, 0.19–0.61%) among those with invasive breast cancer (*p* < 0.05). In addition, among invasive breast cancer patients, the 1 year cumulative events of DVT vein thrombosis were 0.6% (95% CI, 0.3–0.9%), the 2 year events were 0.9% (95% CI, 0.6–1.2%), the 5 year events were 1.2% (95% CI, 0.9–1.6%), and the 10 year events were 1.7% (95% CI, 1.2–2.2%) (Figure 1A). The 1 year cumulative events of PE in invasive breast cancers were 0.4% (95% CI, 0.2–0.6%), the 2 year ones were 0.5% (95% CI, 0.3–0.7%), the 5 year events were 0.7% (95% CI, 0.4–1.0%), and the 10 year events were 0.9% (95% CI, 0.5–1.2%) (Figure 1B). The 1 year cumulative events of VTE in women with invasive breast cancers was 0.9% (95% CI, 0.6–1.3%), the 2 year ones were 1.3% (95% CI, 0.9–1.7%), the 5 year events were 1.8% (95% CI, 1.4–2.3%), and the 10 year events were 2.4% (95% CI, 1.8–3%) (Figure 1C).

### 3.3. Factors Associated with Venous Thromboembolism Occurrence

The highest prevalence of VTE was found in the group of invasive breast cancers other than ductal and lobular. In this group, the 1 year cumulative events of VTE were 1.3% (95% CI, 0–3.1%), the 2 year were 2.0% (95% CI, 0–4.3%), the 5 year were 5.5% (95% CI, 1.4–9.4%), and the 10 year were 5.5% (95% CI, 1.4–9.4%). 

We further investigated the possible factors associated with the occurrence of VTE events. Table 2 shows the factors associated with DVT, PE, or VTE in the whole cohort. In particular, in the univariate analysis, the occurrence of DVT, PE, and VTE seemed associated with woman’s age, BMI, post-menopausal status, chronic hypertension, chronic lung disease, chronic heart failure, type of breast surgery, type of axilla surgery, type of plastic surgery, and breast invasive cancer lesion (Table 2). However, after the multivariate analysis, only the woman’s age, BMI, chronic hypertension, and invasive breast cancer were significantly associated factors (Table 3). 

Table 4 and Table 5 show the factors associated with DVT, PE, or VTE in the invasive cancer sub-cohort. In this case, the following factors seemed to be associated with DVT, PE, and VTE development: woman’s age, BMI, post-menopausal status, chronic hypertension, chronic lung disease, invasive breast cancers other than ductal and lobular histology, adjuvant chemotherapy, tumor size, nodal status, tumor stage, tumor grading, comedo-like necrosis, tumor molecular subtype, lymph node extracapsular invasion, matted axilla lymph nodes, adjuvant radiotherapy, adjuvant chemotherapy, locoregional recurrences, and distant metastasis recurrences. However, after the multivariate analysis, as shown in Table 6, the significantly associated factors with DVT, EP, or VTE were the following: the woman’s age, BMI, chronic hypertension, chronic lung disease, histotypes of invasive carcinoma other than ductal or lobular, TNM stage, comedo-like necrosis, basal-like molecular subtype, matted axilla lymph nodes, locoregional recurrence, distant metastases, adjuvant radiotherapy, and adjuvant chemotherapy.

### 3.4. Overall Survival and Venous Thromboembolism Occurrence

**Figure 2:**
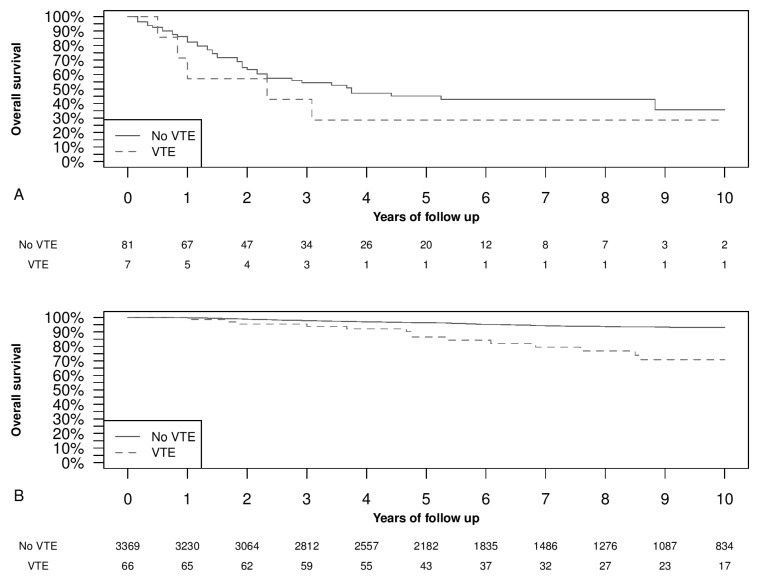
Kaplan–Meier curves of overall survival time divided into presence or absence of venous thromboembolism (VTE) events. Panel (**A**) TNM stage IV (*p* = 0.332). Panel (**B**) TNM stages I, II, and III (*p* < 0.05). The *p*-values refer to log-rank test.

Figure 2 shows the Kaplan–Meier analysis of overall survival in patients who had a stage IV at diagnosis (Figure 2A); and stages I, II, and III at diagnosis (Figure 2B). In Figure 2A the difference was not statistically significant, showing a shorter survival in women with a synchronous VTE. Meanwhile, in Figure 2B, the VTE group presented a significantly shorter survival than women who did not develop VTE (*p* < 0.05). Introducing in a Cox regression the VTE as a time-dependent variable, to avoid the possible immortal-time bias, and adjusting for tumor grade, stage, and histology, the VTE HR for overall survival was 5.3 (95% CI, 3.3–8.6) (*p* < 0.05).

## 4. Discussion

We found a low prevalence of thromboembolic events in the whole cohort of breast surgery patients. In particular, among patients with invasive breast cancer, VTE events occurred in 1.8% of women (95% CI, 1.4–2.3%) at 5 year of follow-up. Thromboembolic events were significantly higher in patients with invasive breast cancer than in women with carcinoma in situ breast cancer or benign breast pathology. Increased BMI, chronic hypertension, chronic lung disease, and invasive breast cancer were significantly associated with VTE events in the whole cohort. In invasive breast cancer sub-cohort, increased BMI and chronic hypertension were significantly associated with DVT or PE. In addition, increased women’s age, TNM stage III–IV, basal-like molecular subtype, distant metastasis occurrence during the follow-up, and adjuvant chemotherapy were significantly associated with DVT. Meanwhile, chronic lung disease, other types of invasive carcinoma, comedo-like necrosis, and matted axilla lymph nodes resulted significantly associated with PE. Moreover, a short survival was observed in women affected by advanced disease and VTE. 

### 4.1. Venous Thromboembolism Occurrence

Concerning VTE incidence, Zammar et al. found a 0% 30 day thromboembolic event rate among patients who underwent conservative breast surgery, concluding that breast conservative surgery may be safely managed without chemical VTE prophylaxis because of the acceptable risk with exclusive mechanical prophylaxis [25]. The main limitations of this study are that it considered only clinically suspected VTE episodes and that it accounted for VTE occurrence only within 30 days after surgery. Other authors considered surgery-related all the events occurring during the first three months from surgery [15]. In a recent article by Momeni et al. that included 52547 breast surgery interventions (including breast conservative surgery and mastectomies), the prevalence of surgery-related VTE events was 0.75% (95% CI 0.68–0.83%) within the first three months from surgery, and most of the events were recorded after discharge [11]. In our population, which also included breast conservative surgery and mastectomies, the cumulative VTE events during the first three months were 0.4% (95% CI 0.19–0.61%), only slightly lower than the values found by Momeni et al. [11]. However, our study considered VTE episodes independently by the time frame of occurrence (regarding more than the first three months of follow-up) and whether they were clinically or radiologically detected. Another recent study by Brand et al. described a VTE incidence of 5.1% at 6 months after diagnosis [26], while Khan et al. found an overall VTE prevalence of 4.63% (95% CI 4.28–5.00%) during a follow-up interval of 13 years [14]. The lower incidence of VTE found in our population in comparison with other studies can be explained by population differences, as well as by the great institutional attention in VTE prophylaxis [11,14,26].

### 4.2. Factors Associated with VTE

In their study, Brand et al. found the following VTE predictive factors: older age, higher weight, history of previous VTE, comorbidities, tumor size greater than 4 cm, progesterone receptor-negative disease, more than four affected nodes, and chemo-endocrine therapy [26]. In our study, we confirmed many of these factors. Among significant factors associated with thromboembolism occurrence in our population, we observed well-known factors, such as high BMI, invasive breast cancer histology, chemotherapy, and distant metastases, all of which have been thoroughly discussed in the literature [5,7,26,27,28]. The interesting results we obtained with this study are related to the fact that some risk factors were associated with VTE occurrence but not with PE development, whereas other factors associated with PE occurrence were shown to be insignificant in the development of VTE. In detail, we observed that comedo-like necrosis is highly associated with PE occurrence, while the basal-like subtype is related to DVT or VTE occurrence. This finding can be explained by the tendency of basal-like tumors to metastasize preferably via blood vessels rather than lymphatic vessels [29], increasing, therefore, the chance of VTE occurrence due to microscopic neoplastic emboli or to the presence of thrombogenic cancer-released molecules in the bloodstream. For the comedo-like necrosis, the mechanistic explanation is not clear because, usually, this histologic feature does not have any significant association with distant metastases [30].

Regarding comorbidities that may influence the occurrence of thromboembolism, while BMI and old age are recognized risk factors for thromboembolic events in general, a recent sizeable study found only a slight correlation between comorbidities and breast cancer for what concerned the thromboembolic event rate [31]. In our opinion, a possible explanation may simply be the more accurate preoperative assessment and prophylactic therapy assumed by this group of patients at a recognized high risk of VTE. Albeit in population cohort studies, an increased blood pressure seemed to be a protective factor against VTE [32], in patients affected by cancer, it was found associated with an increased risk of VTE [27,28]. In our population, we found a significantly increased risk of VTE in patients affected by chronic hypertension, which may be likely mediated by inflammation mechanisms [28].

Lastly, as far as the medical treatment of breast cancer is concerned, our study did demonstrate a correlation between VTE and chemotherapy, concurring with the majority of studies found in the literature. Chemotherapy is correlated with high thrombotic risk, both because chemotherapeutic drugs directly act on endothelial surfaces and because the injection site frequently constitutes a location for VTE development [33,34,35]. Furthermore, some studies found a significant correlation between VTE and hormonal therapy (including tamoxifen) [5,36]. The lack of this last correlation in our setting could be due to a relatively small number of women treated with tamoxifen (less than the 50% of women treated with adjuvant hormonal therapy). In contrast, the majority were treated with aromatase inhibitors, which are notoriously known to be associated with a lower VTE risk [5]. A second explanation could be again the high attention placed in the prevention and treatment of VTE in our center. Moreover, there is still debate about the controversial role of tamoxifen in the possible increase in VTE risk [5,37,38,39]. Furthermore, the influence of chemotherapy, genetic susceptibility, and older age contributed to the increase in VTE risk [36,40]. Additionally, our data confirm that chemotherapy and older age were both two independent factors associated with VTE.

### 4.3. Distant Metastases and Overall Survival

As far as distant metastases are concerned, we observed a higher incidence of VTE events not only in those patients who presented with metastases at diagnosis, but also in women who were subsequently diagnosed with locoregional and distant recurrences during the follow-up. Locally advanced and metastatic breast cancer are commonly recognized to be associated with hypercoagulability and VTE occurrence [7,12,41]. This particular situation could be explained through the presence of circulating tumor cells (CTCs) in the bloodstream of breast cancer patients [42]. In addition, we confirmed the known association between VTE and shorter survival in women affected by distant metastases [7,43]. This association may be partly due to the possible synergy between increased coagulation and CTC intravasation [7]. Starting from this knowledge, the use of coagulation markers as prognostic factors in breast cancer was previously proposed [7,44,45]. In addition, the coagulation system has also been supposed to be a therapeutic target [7]. In particular, increasing evidence points towards an association between tumor progression and platelet function [46,47,48]. The interchange between CTCs and coagulation factors is complex and mutual. Platelet activation and aggregation are implicated in enabling coagulation-mediated metastasis, and tumor-derived cytokines and growth factors are involved in thrombocytosis [49,50]. Moreover, tumor-cell-secreted factors (e.g., tumor-associated tissue factor or thrombin) induce platelet activation and aggregation, thereby shielding tumor cells from high-velocity shear forces and the immune system continuous monitoring [49,51,52]. Upon activation, platelets change morphology and release granular contents (e.g., P-selectin, fibrinogen, or Factors V), facilitating additional adhesion and aggregation [46,53,54]. Some new approaches have been proposed to treat breast cancer, targeting this synergic interaction between platelets and circulating cancer cells. For example, thrombopoietin gene silencing can reduce platelet count and breast cancer progression in animal models [55]. Furthermore, the use of low molecular weight heparin-based nanoparticles to carry doxorubicin increases the therapeutic effect in animal models of metastatic breast cancer [56,57].

Finally, in the current literature, several cases of VTE occurring in the immediate preoperative period are even responsible for the consequent cancer diagnosis [58]. Therefore, an accurate evaluation in VTE is suggested in patients without any apparent risk factor to exclude the most common age- and gender-related cancers, such as breast cancer in the post-menopausal female population.

### 4.4. Strengths and Weaknesses

Both advantages and limitations of our study are primarily related to the retrospective cohort study design characteristics: the number of patients included in the study is somewhat significant, with 5039 women having been diagnosed with breast pathology, and the follow-up program was conducted to cover a period of 10 years (which allowed a thorough analysis of post-surgical and follow-up VTE occurrence). However, the study also presents a minor bias since data from over a decade ago was not always easy to access. Therefore, we were unable to detail the type of VTE management in some cases. Furthermore, the secondary analyses that consider PE and DVT separately should be taken with caution due to the possible overfitting associated with a low number of events, particularly in the PE sub-analysis. Although the ratios in the main analysis and the sub-analysis evolve in the same direction, the limited number of events limits this study’s possibility of arriving at any conclusion in these secondary analyses stratifying for PE and DVT. Another possible limitation is the overestimation of the rate of VTE for long follow-up using the Kaplan–Meier analysis [59]. However, this bias is mainly related to cancers with a relatively high mortality rate. In our case, breast cancer has a relatively low mortality rate, and the cumulative DVT with Kaplan–Meier or a competing risk model were overlapping, letting us use the Kaplan–Meier analysis [23]. Furthermore, the immortality-time bias can affect the assessment of the impact of VTE on mortality [60]. To partially overcome this bias, VTE was included as a time-dependent variable in the Cox model [60].

### 4.5. Generalisability

Furthermore, cohort studies, consisting of patients treated in the everyday clinic, help to capture large sample sizes and allow a better generalizability of results while including a mixed population. Moreover, the population included was treated from 2002 to 2012 to obtain an extended follow-up; however, the time that lasted from the treatment is also a limiting factor for the generalizability of our results because of the evolving management of breast cancer.

### 4.6. Relevance of the Findings and Unanswered Questions

The knowledge about the risk factors of VTE could be helpful as a piece of prognostic information, but also to eventually target preventive treatment for VTE, since the co-existence of invasive breast cancer and VTE has a significant impact on survival. In particular, new evidence suggests, in humans, a beneficial effect of statins in reducing the risk of breast cancer distant metastasis and in reducing the risk of VTE recurrence [61,62,63].

Many issues are not completely clarified, starting from the association between hypercoagulability, CTCs, and VTE. More studies on this argument should be conducted because of the possible implications for the therapy and prognosis.

## 5. Conclusions

In summary, after breast surgery, most VTE events occurred during the first five years of follow-up, with a significantly higher prevalence among invasive breast cancer patients. Furthermore, those VTE events were significantly correlated to women’s age, BMI, chronic hypertension, chronic lung disease, tumor type, stage, comedo-like necrosis, recurrences, adjuvant chemotherapy, and radiation therapy. In addition, VTE occurrence significantly impacted survival in invasive breast cancer patients.

## Figures and Tables

**Table 1:** Population characteristics: (A) considering the whole cohort of women treated with breast surgery; and (B) considering only breast invasive cancers.

Sample	Variables	Values
(A) All the population	Woman age (years)	57.72 (±14.27)
	BMI (kg/m²)	25.87 (±3.94)
	Tobacco smoke	5.04% (254/5039)
	Familial history of cancer	39.17% (378/965)
	Previous estrogen/progesterone use	31.42% (224/713)
	Post-menopausal status	73.09% (3681/5036)
	History of previous VTE	1.61% (81/5039)
	Hypothyroidism	11.33% (571/5039)
	Chronic hypertension	26.35% (1328/5039)
	Chronic lung disease	2.4% (121/5039)
	Chronic heart failure	9.64% (486/5039)
	Breast surgery	
	Conservative	61.38% (3093/5039)
	Mastectomy	38.62% (1946/5039)
	Axilla surgery	75.65% (3812/5039)
	Oncoplastic breast surgery	
	No plastic surgery	76.38% (3849/5039)
	Immediate reconstruction	12.24% (617/5039)
	Delayed reconstruction	11.37% (573/5039)
(B) Considering only breast invasive cancers	Non-surgical therapy	
	Neoadjuvant chemotherapy	5.48% (193/3524)
	Adjuvant radiotherapy	54.57% (1923/3524)
	Adjuvant chemotherapy	39.76% (1401/3524)
	Adjuvant hormonal therapy	74.32% (2619/3524)
	Tumor characteristics	
	Comedo-like necrosis	6.84% (241/3524)
	Multifocality/multicentricity	17.93% (632/3524)
	EIC	21.77% (767/3524)
	PVI	14.25% (502/3524)
	Peritumoral inflammation	2.47% (87/3524)
	Molecular subtype	
	Luminal A	31.84% (1122/3524)
	Luminal B	24.23% (854/3524)
	Luminal Her	5.82% (205/3524)
	Her enriched	4.37% (154/3524)
	Basal-like	8.6% (303/3524)
	Unknown	25.14% (886/3524)
	Lymph node characteristics	
	Isolated tumor cells	2.21% (78/3524)
	Micrometastasis	6.81% (240/3524)
	Extracapsular lymph node invasion	8.97% (316/3524)
	Matted axilla lymph nodes	3.18% (112/3524)
	Recurrences	
	Loco-regional	5.7% (201/3524)
	Distant metastases	7.01% (247/3524)

Acronyms: BMI = body mass index; EIC = extensive intraductal component; PVI = peritumoral vascular invasion; VTE = venous thromboembolism.

**Table 2:** Women characteristics and tumor histology considering the whole cohort of women treated with breast surgery. Univariate Cox proportional hazards model analysis evaluating for a new diagnosis of deep vein thrombosis (DVT), pulmonary embolism (PE), or venous thromboembolism (VTE) after breast surgery and possible associated factors.

Variables	DVT		PE		VTE	
	HR (95% CI)	*p*	HR (95% CI)	*p*	HR (95% CI)	*p*
Woman’s age (years)	1.04 (1.02–1.06)	<0.05	1.02 (0.99–1.05)	0.214	1.03 (1.01–1.05)	<0.05
BMI (kg/m²)	1.08 (1.02–1.14)	<0.05	1.13 (1.06–1.2)	<0.05	1.1 (1.05–1.15)	<0.05
Tobacco smoke	1.24 (0.38–3.98)	0.722	0.71 (0.1–5.2)	0.733	0.83 (0.26–2.63)	0.750
Familial history of cancer	0.44 (0.09–2.14)	0.311	0.79 (0.07–8.69)	0.846	0.52 (0.14–1.92)	0.325
Previous estrogen/progesterone use	0.86 (0.17–4.45)	0.861	1.43 (0.24–8.55)	0.696	1.09 (0.33–3.6)	0.894
Post-menopausal status	4.16 (1.49–11.57)	<0.05	3.01 (0.91–10)	0.072	3.4 (1.56–7.43)	<0.05
Diabetes mellitus	0.81 (0.2–3.34)	0.773	0.72 (0.1–5.31)	0.748	0.84 (0.26–2.67)	0.765
Hypothyroidism	1.28 (0.57–2.85)	0.550	2.14 (0.86–5.3)	0.101	1.55 (0.83–2.89)	0.166
Chronic hypertension	3.78 (2.13–6.71)	<0.05	4.61 (2.11–10.07)	<0.05	4.33 (2.68–7.01)	<0.05
Chronic lung disease	0.89 (0.12–6.49)	0.912	5.26 (1.58–17.46)	<0.05	1.9 (0.6–6.03)	0.278
Chronic heart failure	1.62 (0.73–3.62)	0.236	2.17 (0.82–5.73)	0.118	1.97 (1.06–3.67)	<0.05
Breast surgery						
Conservative	Reference	---	Reference	---	Reference	---
Mastectomy	1.39 (0.79–2.45)	0.256	2.06 (0.97–4.41)	0.062	1.56 (0.97–2.49)	0.065
Axilla surgery						
No axilla surgery	0.08 (0.01–0.57)	<0.05	0.17 (0.02–1.37)	0.097	0.12 (0.03–0.5)	<0.05
SLNB	Reference	---	Reference	---	Reference	---
CALND	1.26 (0.7–2.28)	0.438	1.78 (0.77–4.1)	0.175	1.54 (0.93–2.55)	0.092
Oncoplastic breast surgery						
No plastic surgery	Reference	---	Reference	---	Reference	---
Immediate reconstruction	1.45 (0.67–3.14)	0.340	0.28 (0.04–2.09)	0.215	1.05 (0.52–2.13)	0.892
Delayed reconstruction	1.13 (0.47–2.69)	0.785	1.17 (0.4–3.4)	0.769	0.97 (0.46–2.04)	0.932
History of previous VTE	1.31 (0.18–9.5)	0.789	2.37 (0.32–17.45)	0.398	1.82 (0.45–7.45)	0.402
Histology						
Negative	Reference	---	Reference	---	Reference	---
Ductal in situ carcinoma	0 (0–Inf) (*)	0.996	0 (0–Inf) (*)	0.997	0 (0–Inf) (*)	0.995
Invasive carcinoma non-special type	13.77 (1.89–100.49)	<0.05	7.47 (1–55.82)	<0.05	9.87 (2.4–40.56)	<0.05
Lobular invasive carcinoma	14.2 (1.71–117.92)	<0.05	4.71 (0.43–51.99)	0.206	9.47 (2.01–44.61)	<0.05
Ductal and lobular invasive carcinoma	8.45 (0.77–93.21)	0.081	4.29 (0.27–68.69)	0.303	6.38 (1.07–38.21)	<0.05
Other type of invasive carcinoma	28.1 (3.14–251.38)	<0.05	28.58 (3.19–255.73)	<0.05	24.98 (5.19–120.25)	<0.05

(*) No case of DVT, PE or VTE was registered during the considered follow-up period. Other acronyms: BMI = body mass index; CALND = complete axillary lymph node dissection; CI = confidence interval; HR = hazard ratio; SLNB = sentinel lymph node biopsy.

**Table 3:** The table shows the factors associated with a new diagnosis of deep vein thrombosis (DVT), pulmonary embolism (PE), or venous thromboembolism (VTE) after breast surgery. In addition, the table shows the whole cohort analysis. The reported values refer to multivariate Cox proportional hazards model analysis considering the new diagnosis of DVT, PE, or VTE after breast surgery.

Variables	DVT		PE		VTE	
	HR (95% CI)	*p*	HR (95% CI)	*p*	HR (95% CI)	*p*
All breast surgery cohort (*)						
Woman’s age (years)	1.02 (1–1.05)	0.080				
BMI (kg/m²)	1.06 (1–1.12)	<0.05	1.10 (1.03–1.17)	<0.05	1.07 (1.02–1.11)	<0.05
Chronic hypertension	2.10 (1.13–3.89)	<0.05	2.84 (1.26–6.39)	<0.05	2.83 (1.72–4.65)	<0.05
Invasive breast cancer	13.55 (1.84–99.54)	<0.05	8.03 (1.07–60.15)	<0.05	10.71 (2.6–44.11)	<0.05

The initial multivariate model considered the following factors: (*) woman’s age, BMI, post-menopausal status, chronic hypertension, chronic lung disease, chronic heart failure, type of breast surgery, type of axilla surgery, type of plastic surgery, and breast lesion histology. Other acronyms: BMI = body mass index; CI = confidence interval; HR = hazard ratio; TNM = tumor, nodes, and metastases.

**Table 4:** Women characteristics and tumor histology considering only the sub-cohort of invasive breast tumors. Univariate Cox proportional hazards model analysis considering for new diagnosis of deep vein thrombosis (DVT), pulmonary embolism (PE), or venous thromboembolism (VTE) after breast surgery and possible associated factors.

Variables	DVT		PE		VTE	
	HR (95% CI)	*p*	HR (95% CI)	*p*	HR (95% CI)	*p*
Woman’s age (years)	1.03 (1.01–1.05)	<0.05	1 (0.98–1.03)	0.785	1.02 (1–1.04)	0.065
BMI (kg/m²)	1.07 (1.02–1.13)	<0.05	1.12 (1.05–1.2)	<0.05	1.09 (1.05–1.14)	<0.05
Tobacco smoke	1.01 (0.93–1.1)	0.751	0.75 (0.37–1.52)	0.422	0.99 (0.91–1.07)	0.754
Familial history of cancer	0.45 (0.09–2.16)	0.319	0.79 (0.07–8.7)	0.847	0.52 (0.14–1.93)	0.332
Previous estrogen/progesterone use	1 (0.19–5.17)	0.997	1.69 (0.28–10.14)	0.564	1.27 (0.38–4.22)	0.695
Post-menopausal status	2.78 (1–7.75)	0.050	1.97 (0.59–6.56)	0.269	2.25 (1.03–4.93)	<0.05
Diabetes mellitus	0.67 (0.16–2.77)	0.583	0.61 (0.08–4.47)	0.622	0.7 (0.22–2.22)	0.545
Hypothyroidism	1.13 (0.5–2.51)	0.771	1.94 (0.78–4.83)	0.155	1.38 (0.74–2.58)	0.308
Chronic hypertension	2.6 (1.46–4.63)	<0.05	3.65 (1.63–8.2)	<0.05	3.13 (1.92–5.11)	<0.05
Chronic lung disease	0.72 (0.1–5.22)	0.745	4.3 (1.29–14.32)	<0.05	1.54 (0.48–4.89)	0.467
Chronic heart failure	1.21 (0.54–2.7)	0.640	1.65 (0.62–4.37)	0.315	1.48 (0.8–2.77)	0.215
Breast surgery						
Conservative	Reference	---	Reference	---	Reference	---
Mastectomy	0.98 (0.55–1.74)	0.942	1.52 (0.7–3.32)	0.288	1.11 (0.69–1.79)	0.656
Axilla surgery						
SLNB	Reference	---	Reference	---	Reference	---
CALND	1.09 (0.6–1.96)	0.775	1.55 (0.67–3.56)	0.305	1.33 (0.81–2.2)	0.264
Oncoplastic breast surgery						
No plastic surgery	Reference	---	Reference	---	Reference	---
Immediate reconstruction	1.07 (0.49–2.31)	0.866	0.21 (0.03–1.56)	0.128	0.78 (0.38–1.58)	0.486
Delayed reconstruction	0.96 (0.4–2.3)	0.930	1.02 (0.35–2.98)	0.969	0.83 (0.4–1.76)	0.631
History of previous VTE	1 (0.14–7.23)	0.997	1.83 (0.25–13.48)	0.555	1.4 (0.34–5.71)	0.641
Histology						
Invasive carcinoma non-special type	Reference	---	Reference	---	Reference	---
Lobular invasive carcinoma	1.03 (0.43–2.45)	0.946	0.63 (0.15–2.7)	0.533	0.96 (0.45–2.02)	0.912
Ductal and lobular invasive carcinoma	0.62 (0.15–2.56)	0.504	0.58 (0.08–4.32)	0.594	0.65 (0.2–2.08)	0.468
Other type of invasive carcinoma	2.04 (0.72–5.74)	0.177	3.8 (1.29–11.19)	<0.05	2.52 (1.14–5.57)	<0.05

Other acronyms: BMI = body mass index; CALND = complete axillary lymph node dissection; CI = confidence interval; HR = hazard ratio; SLNB = sentinel lymph node biopsy.

**Table 5:** Tumor characteristics and tumor recurrences considering only invasive breast tumors. Univariate Cox proportional hazards model analysis considering for new diagnosis of deep vein thrombosis (DVT), pulmonary embolism (PE), or venous thromboembolism (VTE) after breast surgery and possible associated factors.

Variables	DVT		PE		VTE	
	HR (95% CI)	*p*	HR (95% CI)	*p*	HR (95% CI)	*p*
Tumor staging						
Tumor size (T3 or T4)	2.6 (1.03–6.58)	<0.05	3.9 (1.34–11.34)	<0.05	3.3 (1.63–6.66)	<0.05
Nodal status (N2 or N3)	3.59 (1.94–6.64)	<0.05	1.35 (0.46–3.93)	0.580	2.94 (1.73–5)	<0.05
Tumor grading (G3)	1.63 (0.9–2.95)	0.108	2.48 (1.15–5.35)	<0.05	1.9 (1.17–3.08)	<0.05
TNM stage (III or IV)	3.57 (1.98–6.44)	<0.05	2.06 (0.87–4.92)	0.102	3.11 (1.89–5.12)	<0.05
Tumor characteristics						
Comedo-like necrosis	0.6 (0.15–2.47)	0.480	4.12 (1.65–10.26)	<0.05	1.57 (0.72–3.43)	0.259
Multifocality/multicentricity	1.36 (0.69–2.68)	0.370	1.66 (0.7–3.95)	0.251	1.38 (0.79–2.42)	0.259
EIC	0.67 (0.31–1.43)	0.295	1.48 (0.64–3.41)	0.356	0.93 (0.52–1.65)	0.800
PVI	0.97 (0.41–2.29)	0.950	1.18 (0.41–3.44)	0.755	1.14 (0.58–2.23)	0.708
Peritumoral inflammation	1.68 (0.41–6.94)	0.471	0 (0–Inf) (*)	0.997	1.15 (0.28–4.69)	0.848
Molecular subtype (Basal-like)	3.15 (1.57–6.34)	<0.05	2.07 (0.71–6.02)	0.180	2.47 (1.33–4.62)	<0.05
Lymph nodes characteristics						
Non axillary locoregional lymph nodes	1.54 (0.21–11.19)	0.668	0 (0–Inf) (*)	0.996	1.06 (0.15–7.6)	0.957
Isolated tumor cells	0 (0–Inf) (*)	0.996	3.52 (0.83–14.89)	0.087	1.27 (0.31–5.18)	0.740
Micrometastasis	0.94 (0.29–3.01)	0.912	2.51 (0.86–7.27)	0.091	1.59 (0.73–3.47)	0.247
Extracapsula invasion	2.75 (1.33–5.7)	<0.05	2.07 (0.71–6.03)	0.180	2.73 (1.49–5)	<0.05
Matted axilla lymph nodes	2.52 (0.78–8.14)	0.121	6.56 (2.26–19.09)	<0.05	4.24 (1.94–9.28)	<0.05
Recurrences						
Locoregional recurrence	1.01 (0.31–3.27)	0.982	2.79 (0.96–8.1)	0.060	1.73 (0.79–3.79)	0.170
Distant metastases	6.98 (3.77–12.91)	<0.05	3.49 (1.31–9.27)	<0.05	5.79 (3.4–9.84)	<0.05
Non-surgical therapies						
Neoadjuvant chemotherapy	0.76 (0.18–3.12)	0.700	2.23 (0.67–7.45)	0.190	1.35 (0.54–3.36)	0.515
Adjuvant radiotherapy	1.71 (0.93–3.16)	0.086	0.69 (0.32–1.48)	0.337	1.29 (0.79–2.11)	0.301
Adjuvant chemotherapy	2.41 (1.34–4.35)	<0.05	1.5 (0.7–3.24)	0.299	2.02 (1.25–3.26)	<0.05
Adjuvant hormonal therapy	1.07 (0.54–2.1)	0.847	0.62 (0.27–1.38)	0.242	0.91 (0.53–1.56)	0.729
Tamoxifen	0.76 (0.42–1.4)	0.382	0.8 (0.36–1.8)	0.589	0.76 (0.46–1.26)	0.292

(*) No case of DVT, PE or VTE was registered during the considered follow-up period. Other acronyms: CI = confidence interval; HR = hazard ratio.

**Table 6:** The table shows the factors associated with a new diagnosis of deep vein thrombosis (DVT), pulmonary embolism (PE), or venous thromboembolism (VTE) after breast surgery. In addition, the table shows the sub-cohort of invasive breast cancer analysis. The reported values refer to multivariate Cox proportional hazards model analysis considering the new diagnosis of DVT, PE, or VTE after breast surgery.

Variables	DVT		PE		VTE	
	HR (95% CI)	*p*	HR (95% CI)	*p*	HR (95% CI)	*p*
Invasive breast cancer sub-cohort (†)						
Woman’s age (years)	1.04 (1.01–1.07)	<0.05				
BMI (kg/m²)	1.06 (1.01–1.12)	<0.05	1.10 (1.03–1.17) (¶)	<0.05	1.06 (1.00–1.12) (¶)	0.054
Chronic hypertension	1.98 (1.07–3.63)	<0.05	2.99 (1.31–6.85) (¶)	<0.05	2.35 (1.3–4.24) (¶)	<0.05
Chronic lung disease			2.93 (0.87–9.90) (§)	0.084		
Other type of invasive carcinoma			3.5 (1.18–10.39) (§)	<0.05	1.93 (0.69–5.4) (§)	0.211
TNM stage (III–IV)	1.91 (1–3.65)	0.050			3.63 (2.01–6.55) (§)	<0.05
Comedo-like necrosis			5.24 (2.08–13.19) (§)	<0.05	0.69 (0.17–2.86) (§)	0.612
Molecular subtype (basal-like)	2.46 (1.19–5.08)	<0.05			3.56 (1.76–7.18) (§)	<0.05
Matted axilla lymph nodes			7.4 (2.53–21.69) (§)	<0.05		
Locoregional recurrence	0.44 (0.13–1.51)	0.193	2.89 (0.99–8.42) (§)	0.052		
Distant metastases diagnosed during follow-up	4.42 (2.17–8.99)	<0.05			7.04 (3.8–13.04) (§)	<0.05
Adjuvant radiotherapy	1.74 (0.92–3.26)	0.086				
Adjuvant chemotherapy	2.11 (1.02–4.37)	<0.05				

The initial multivariate model considered the following factors: (†) Woman’s age, BMI, post-menopausal status, chronic hypertension, chronic lung disease, breast lesion histology, adjuvant chemotherapy, tumor size, nodal status, tumor grading, tumor stage, comedo-like necrosis, tumor molecular subtype, lymph node extracapsular invasion, matted axilla lymph nodes, adjuvant radiotherapy, adjuvant chemotherapy, locoregional recurrences, and distant metastasis recurrences. (§) Multivariate model with adjustment for BMI and chronic hypertension. (¶) Model including BMI and chronic hypertension. Other acronyms: BMI = body mass index; CI = confidence interval; HR = hazard ratio; TNM = tumor, nodes, and metastases.

## Data Availability

The data that support the findings of this study are available, but restrictions apply to the availability of these data, which was used under license for the current study, and so are not publicly available. Data are, however. available from the authors upon reasonable request and with permission of the Internal Review Board.

## Supplementary Materials

The following supporting information can be downloaded at: https://www.mdpi.com/article/10.3390/cancers14040988/s1, Table S1: Breast cancer staging.
