# Supplementary material for: Incidence and Risk Factors for Venous Thromboembolism in Female Patients Undergoing Breast Surgery
Source: Cancers (Basel). 2022 Feb 16;14(4):988. doi: 10.3390/cancers14040988 (PMC8870485; doi:10.3390/cancers14040988)
Supplement: Supplementary file 1 [file cancers-14-00988-s001.zip › cancers-1563579-supplementary.pdf]

cancers

MDPI

Supplementary Materials: Incidence and Risk Factors for Venous Thromboembolism in Female Patients Undergoing Breast Surgery

Ambrogio P. Londero, Serena Bertozzi, Carla Cedolini, Silvia Neri, Michela Bulfoni, Maria Orsaria, Laura Mariuzzi, Alessandro Uzzau, Andrea Risaliti and Giovanni Barillari

Table S1. Breast cancer staging.

Variables	Values
Tumor Size	
Tis	11.55% (460/3983)
T1	62.72% (2498/3983)
T2	21.04% (838/3983)
T3	2.21% (88/3983)
T4	2.49% (99/3983)
Nodal status	
N0	69.65% (2774/3983)
N1	18.75% (747/3983)
N2	6.18% (246/3983)
N3	5.42% (216/3983)
TNM stage	
Stage 0	11.55% (460/3983)
Stage I	46.55% (1854/3983)
Stage II	27.12% (1080/3983)
Stage III	12.58% (501/3983)
Stage IV	2.21% (88/3983)
Tumor grading (*)	
G 1	12.8% (451/3524)
G 2	60.93% (2147/3524)
G 3	26.28% (926/3524)

(*) Only invasive breast cancer. Acronyms: TNM= tumor, nodes, and metastases.
